# A multiplex serologic platform for diagnosis of tick-borne diseases

**DOI:** 10.1038/s41598-018-21349-2

**Published:** 2018-02-16

**Authors:** Rafal Tokarz, Nischay Mishra, Teresa Tagliafierro, Stephen Sameroff, Adrian Caciula, Lokendrasingh Chauhan, Jigar Patel, Eric Sullivan, Azad Gucwa, Brian Fallon, Marc Golightly, Claudia Molins, Martin Schriefer, Adriana Marques, Thomas Briese, W. Ian Lipkin

**Affiliations:** 1 Center for Infection and Immunity, Mailman School of Public Health, Columbia University, New York, NY USA; 2 Roche Sequencing Solutions, Madison, WI USA; 3 Department of Biology, Farmingdale State College, Farmingdale, NY USA; 4 Lyme and Tick-borne Diseases Research Center, Columbia University, New York, NY USA; 5 Department of Pathology, Stony Brook University, New York, NY USA; 6 Division of Vector-Borne Diseases, Centers for Disease Control and Prevention, Fort Collins, CO USA; 7 Laboratory of Clinical Immunology and Microbiology, National Institute of Allergy and Infectious Diseases, National Institutes of Health, Bethesda, MD USA

## Abstract

Tick-borne diseases are the most common vector-borne diseases in the United States, with serology being the primary method of diagnosis. We developed the first multiplex, array-based assay for serodiagnosis of tick-borne diseases called the TBD-Serochip. The TBD-Serochip was designed to discriminate antibody responses to 8 major tick-borne pathogens present in the United States, including *Anaplasma phagocytophilum, Babesia microti*, *Borrelia burgdorferi*, *Borrelia miyamotoi*, *Ehrlichia chaffeensis*, *Rickettsia rickettsii*, Heartland virus and Powassan virus. Each assay contains approximately 170,000 12-mer linear peptides that tile along the protein sequence of the major antigens from each agent with 11 amino acid overlap. This permits accurate identification of a wide range of specific immunodominant IgG and IgM epitopes that can then be used to enhance diagnostic accuracy and integrate differential diagnosis into a single assay. To test the performance of the TBD-Serochip, we examined sera from patients with confirmed Lyme disease, babesiosis, anaplasmosis, and Powassan virus disease. We identified a wide range of specific discriminatory epitopes that facilitated accurate diagnosis of each disease. We also identified previously undiagnosed infections. Our results indicate that the TBD-Serochip is a promising tool for a differential diagnosis not available with currently employed serologic assays for TBDs.

## Introduction

Tick-borne diseases (TBDs) are the most common vector-borne diseases in the US^1^. As of 2017, 19 bacterial, protozoan and viral agents have been implicated in TBDs^2^. *Borrelia burgdorferi*, the causative agent of Lyme disease, alone accounts for an estimated 300,000 annual cases of TBDs^3–5^. Each year, approximately 3 million clinical specimens are tested for TBDs in the US with serology being the mainstay of TBD diagnosis^6^. The most frequently employed serologic assays for TBD diagnoses include the enzyme-linked immunosorbent assay (ELISA), indirect immunofluorescent assay (IFA), and western blot^6–9^. The diagnostic accuracy of these assays may be affected by a variety of intrinsic limitations that can impede effective diagnosis. For Lyme disease, the recommended method for diagnosis is a two-tiered testing algorithm consisting of an ELISA followed by supplemental western blots^9^. Although this method is specific and sensitive in disseminated disease, its utility in the early phase of disease is limited. It accurately identifies <40% of patients with early disease and can result in up to 28% of IgM western blots yielding false positive results^10,11^. This method is also labor-intensive, and uses subjective criteria for interpretation of results that may impact reproducibility^12,13^. IFA is recommended for diagnosis of tick-borne infections with *Babesia*, *Anaplasma*, *Ehrlichia* and *Rickettsia*. The accuracy of IFAs can vary widely among testing laboratories primarily due to the lack of standardized antigenic targets, crossreactivity, and subjective establishment of positivity thresholds^14^. For other tick-borne agents, specific serologic tests are not yet available, or in the case of Powassan virus or Heartland virus, are only performed in specialized laboratories^15,16^. An additional challenge is the lack of assays that simultaneously test for exposure to the full spectrum of tick-borne agents. This is an important limitation in TBD diagnosis in light of surveillance studies that indicate ticks can harbor more than one human pathogen^17–20^.

The limitations of current platforms have prompted the exploration of alternative diagnostic tools for TBDs. A promising approach for resolving the intrinsic lack of specificity associated with protein assays is the use of recombinant antigens or synthetic peptides that display immunodominant epitopes^21–31^. This strategy improves assay specificity by eliminating non-specific and potentially cross-reactive epitopes. Targeting a combination of such antigens can enhance assay sensitivity and has been shown to improve the diagnosis of early Lyme disease^32–34^. Employing this approach, we established the first highly-multiplexed, array-based assay for diagnostic serology of TBDs called the Tick-borne disease Serochip (TBD-Serochip). The TBD-Serochip offers substantial improvement in target selection by employing an extensive range of linear peptides that identify key specific immunodominant epitopes. Employed in concert on the TBD-Serochip, these peptides can be used to enhance diagnostic accuracy and provide a differential diagnosis not available with currently employed serologic assays for TBDs.

## Results

### TBD-Serochip design

The array slide we employed can accommodate up to 3 million 12-mer linear peptides that can can be divided into 12 subarrays, each containing approximately 170,000 12-mer peptides. Because peptides are programmed for synthesis *in situ*, the array composition can be continuously and inexpensively modified based on performance or the need to address new immunological targets. We exploited this platform to develop a TBD-Serochip that targets 8 principal tick-borne pathogens present in the US, including *Anaplasma phagocytophilum* (agent of human granulocytic anaplasmosis), *Babesia microti* (babesiosis), *Borrelia burgdorferi* (Lyme disease), *Borrelia miyamotoi*, *Ehrlichia chaffeensis* (human monocytic ehrlichiosis), *Rickettsia rickettsii* (Rocky Mountain spotted fever), Heartland virus and Powassan virus. We also included Long Island tick rhabdovirus, a novel virus we identified in *Amblyomma americanum* ticks through metagenomic sequencing^35^ (Table 1).

**Table 1:** Composition of the TBD-Serochip.

Agent and antigens	Number of 12-mer peptides
***Anaplasma phagocytophilum*** MSP2, MSP4, MSP5, P55, P62, Omp1N	16,787
***Babesia microti*** SA-1, BMN1 (-2, -3, -4-5-6, -7, -8, -10, -11, -12-13, -17, -20, -21), GPI 12, AMA1; Hsp70, DnaK, Bmp32	11,333
***Borrelia burgdorferi*** OspA, OspB, OspC, OspD, VlsE, DbpA and B, BmpA-BmpD, P100, OppA, OppA2, RevA, P66, LA7, BBK07, BBK32, BBK50, FlaA, FlaB, FliL, FlgE, DnaK, BBA04, BBA36, BBA57, BBA64, BBA65, BBA66, BBA68, BBA69, BBA73, BBA74, BBI38, BBI42, BBE31, OspE, OspF, Erp (all paralogs), Mlp (all paralogs), Bdr (all paralogs), BBO03 (all paralogs)	91,338
***Borrelia miyamotoi*** GlpQ, FhbA, ipA, P66, OppA2, FlgG, FlaB, FliL, VLP (1, A1, A2, C1, C2, C3, D1, D2, D3, D4, D5, D5S, D6S, D6, D7S, D8, D9, D10, 3 S, A2S, 4 S 15/16, 18), VSP (1,2, 3, 4, 6)	23,946
***Ehrlichia chaffeensis*** P156, P120, P28/omp-1, Gp47, VLPT, SP-related protein	4,156
***Rickettsia rickettsii*** OmpA, OmpB, OmpW, Porin 4, adr1, adr2	5,855
**Heartland virus** N, Gn, Gc, L	4,153
**Powassan virus** polyprotein	7,688
**Long Island tick rhabdovirus** N, P, M, G, L	3,949

We established a database of overlapping 12-mer peptides that tiled the proteome of each of these agents. The decision to use 12-mers was based on the evidence that serum antibodies bind linear epitopes with peptide sequences ranging from 5 to 9 amino acid (aa) and bind most efficiently when targets are flanked by additional aa^36^. For each antigen selected, we downloaded all available protein sequences from the NCBI protein database. Sequences were aligned and used to design 12-mer peptides that tiled each protein with a 11-aa overlap to the preceding peptide in a sliding window pattern. Redundant 12-mers were excluded prior to synthesis. For viral agents, 12-mer peptides were designed that tiled the entire proteome (Table 1). For non-viral agents, antigen selection was more specific, and based on the current understanding of the triggers of humoral immunity for each agent. For *B. burgdorferi*, we selected 62 proteins (including paralogs) that are known to elicit an antibody response in humans^26,37^. For *B. miyamotoi*, serological diagnosis is typically performed using the glycerophosphodiester phosphodiesterase, as this antigen is present in *B. miyamotoi* infection but not *B. burgdorferi*^38–40^. Multiple differentially expressed variable membrane proteins have also been shown to be antigenic in humans, with variable small protein 1, in particular, being highly immunogenic in the early stage of infection^41^. The *B. microti* genome contains one species-specific multi-gene family (*bmn*) that codes for antigenic surface proteins that have been shown to be a reliable tool for *B. microti* detection^42–45^. *A. phagocytophilum* major surface proteins (MSP) have been implicated as the primary diagnostic antigens for anaplasmosis^46^. For both *E. chaffeensis* and *R. rickettsii*, we selected surface proteins historically used for antibody detection^47,48^. For each antigen we included every genetic variant for the 12-mer design. For the OspC antigen of *B. burgdorferi* alone, we designed 12-mer peptides for 25 distinct OspC types. The final design of the TBD-Serochip consisted of 169,205 peptides (Table 1).

### Sample dilution optimization

To test the utility of the TBD-Serochip, we used well-characterized sera and CSF containing antibodies to one or more agents of TBDs including *A. phagocytophilum*, *B. burgdorferi*, *B. microti*, *E. chaffeensis*, and Powassan virus (Table 2). To determine the optimal serum dilution, we tested 16 sera at dilutions of 1:50 and 1:200 and two sera at dilutions of 1:20, 1:50, and 1:200. The 1:50 serum dilution was determined to be optimal for the TBD-Serochip as the 1:200 dilution did not achieve the required sensitivity in early disease samples and the 1:20 dilution generated adverse signal to noise ratio. A 1:5 dilution was used for CSF.

**Table 2:** Samples tested on the TBD-Serochip.

Disease	Sample type	Number of samples tested
Lyme disease	serum	36
Lyme disease - neuroborreliosis	serum	10
Lyme disease - neuroborreliosis	CSF	10
Lyme disease and babesiosis	serum	10
Babesiosis	serum	1
Anaplasmosis	serum	7
Powassan virus disease	serum	6
Ehrlichiosis	serum	1
CDC validation set	serum	32
Southern tick-associated rash illness	serum	10
Southern tick-associated rash illness	CSF	10
Lyme disease negative	serum	15
Negative control - polyomavirus	serum	2
Total samples		150

### Identification and selection of reactive peptides

**Figure 1:**
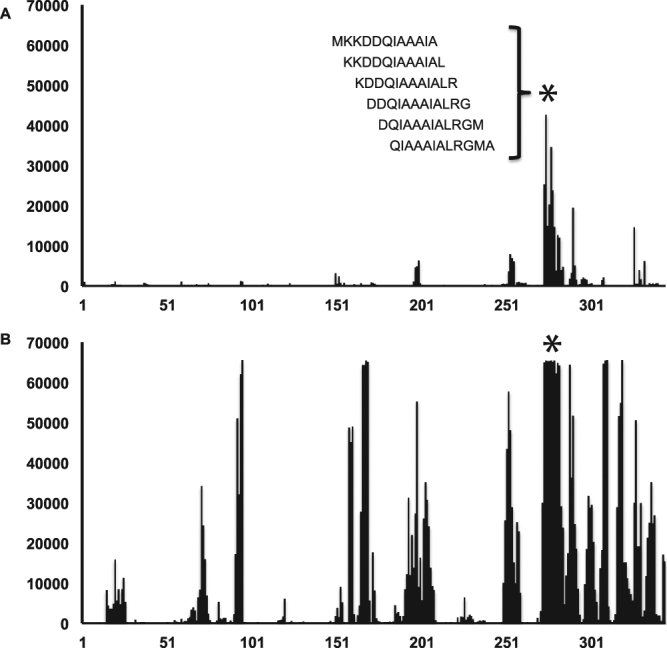
Epitope identification using the TBD-Serochip. Shown is the reactivity data generated for *B. burgdorferi* VlsE antigen using patient sera from early acute Lyme disease (panel A) and neuroborreliosis (panel B). The X-axis represents the location of 12-mer peptides positioned along the contiguous protein sequence of VlsE (corresponding to accession number CAJ41626); Y axis represents the intensity of the fluorescent signal of each peptide. The asterisk specifies the location of the shown 12-mer peptides that make up the major immunoreactive region of the C6 region.

For each agent-positive sample examined, we catalogued all immunoreactive linear epitopes (Fig. 1). To assess reproducibility, we tested 3 sera in duplicate on the same array, and 3 sera on different arrays. The data were highly reproducible with minimal intra- and interchip variation (Supplementary Fig. 1). Reactive 12-mer peptides displayed 85–100% signal intensities relative to background when serum samples were tested on the same or different arrays. We contrasted the immunoreactivity of these epitopes with that of controls (consisting of TBD-negative samples and samples with other TBDs). Any identified epitope that was non-reactive within the control group was considered agent-specific. Our focus was on identification of epitopes that employed in concert would enhance diagnostic sensitivity and specificity of early disease. Thus, we characterized and sorted all agent-specific epitopes by signal intensity, reproducibility, and their utility for diagnosis of disease stage. We selected optimal epitopes that best fulfilled all three criteria.

### Lyme disease

We tested 66 samples from patients with Lyme disease (Table 2, Supplementary Tables 1 and 2). These included 27 sera from early acute Lyme disease (western blot IgM positive, western blot IgG negative), 19 western blot IgG positive sera, and 10 paired sera and CSF from patients diagnosed with acute neuroborreliosis. 10 of these samples (7 IgM positive, 3 IgG positive) previously also tested positive for antibodies to *B. microti*. For controls, we examined 15 sera submitted for Lyme disease testing that were negative by the two-tiered testing algorithm, two sera from patients with a chronic polyomavirus infection, and 10 paired sera and CSF from patients diagnosed with southern tick-associated rash illness, a tick-borne syndrome of unknown etiology^49^. Our analysis resulted in the identification of >100 reactive epitopes including 10 that were most useful for diagnosis of early Lyme disease (Table 3, Supplementary Tables 1 and 2). VlsE was the most prominent antigen in both early and late disease with multiple reactive epitopes identified throughout this lipoprotein (Fig. 1, Table 4). The most consistently reactive epitope was a 17 aa portion of the well-characterized immunodominant C6 region^28,29,50^. This epitope was reactive in every IgG positive Lyme disease serum tested and in 16 out of 27 early Lyme disease samples. This finding underscores the utility of the C6 ELISA for Lyme disease diagnosis. Other frequently reactive epitopes in early Lyme disease were located within FlaB, OspC and BBK07 (Table 3). Although highly reactive in Lyme disease patients, the extensive cross-reactivity of FlaB makes it unsuitable as a diagnostic antigen^51^. The 13 amino acid peptide fragment identified by the TBD-Serochip excludes other cross-reactive epitopes and highlights its diagnostic utility. A similar peptide has been recently employed by Lahey and colleagues to enhance early Lyme disease diagnosis^33^. The OspC Nt epitope along with epitopes located within variable OspC regions were useful for detection of IgM in early disease. An epitope similar to the BBK07 epitope was previously described by Coleman and colleagues^25^. In sera where reactivity to these epitopes was not detected, reactivity to epitopes on other antigens was used for diagnosis, including previously unrecognized epitopes on P100, OppA, Bdr, and paralogs of BBO03, a cp32-encoded lipoprotein. This panel of 10 epitopes was also useful for diagnosis of IgG positive samples (Table 4, Supplementary Table 2).

**Table 3:** Optimal discriminatory linear epitopes identified on the TBD-Serochip.

Antigen (*B. burgdorferi*)	Epitope sequence	Accession number (coordinates)	Antibody class target
VlsE	MKKDDQIAAAIALRGMA	CAJ41626 (274–290)	IgG
FlaB	VQEGVQQEGAQQP	NC001318 (211–221)	IgM and IgG
OspC Nt*	NSGKDGNTSANSA	NP_047005 (20–33)	IgM
OspC typeK	KAILTTDAAKDKG	CAA59253 (132–144)	IgM and IgG
OspC type M	AAILKTNGTKDKG	AC94173 (133–145)	IgM and IgG
P100	SDIDIDSLVTDKVVAA	CAA54797 (199–214)	IgG
BBK07	SEKITKLTPEELENLAK	NC001855 (52–68)	IgG
Bdr	DLENLEKQFDIKFD	ACK75301 (46–53)	IgG
OppA	VAYNMYINGELDFL	AAB97671 (242–255)	IgG
BBO03	KFDKLENHPFLGYPYK	ACL33989 (55–70)	IgG
**Antigen (** ***B. microti*** **)**	**Epitope sequence**	**Accession number (coordinates)**	**Antibody class target**
SA-1 (BMN 1–9)	VLSAGGSGGNGGNG	XP 012648767 (24–37)	IgG
SA-1 (BMN 1–9)	HQEQNNANDSSNPTG	XP 012648767 (40–54)	IgG
SA-1 (BMN 1–9)	EKNKKFNENLVKIEKR	XP 012648767 (118–133)	IgG
BMN 1–17	GENDIIQPPWEDTAP	AF68253 (43–57)	IgG
BMN 1–17	NKSEKAERKSHDTQT	AF68253 (138–152)	IgG
BMN 1–3	GTGWPSEAGGPSEAGG	AAF68236 (59–74)	IgG
**Antigen (** ***A. phagocytophilum*** **)**	**Epitope sequence**	**Accession number (coordinates)**	**Antibody class target**
MSP2 (P44)	FDWNTPDPRIGFKDNML	WP_044108210 (75–91)	IgG
MSP2 (P44)	TSGKDIVQFAKAVEIS	WP_044108210 (160–175)	IgG
**Antigen (Powassan virus)**	**Epitope sequence**	**Accession number (coordinates)**	**Antibody class target**
Glycoprotein	EDLALPWKHKDNQDWN	ADK37753 (499–514)	IgG

**Table 4:** List of most frequently reactive IgG epitopes in samples from patients with acute neuroborreliosis.

Antigen	Peptide	NB1		NB2		NB3		NB4		NB5		NB6		NB7		NB8		NB9		NB10	
		CSF	Serum	CSF	Serum	CSF	Serum	CSF	Serum	CSF	Serum	CSF	Serum	CSF	Serum	CSF	Serum	CSF	Serum	CSF	Serum
VlsE	MKKDDQIAAAIALRGMA	+	+	+	+	+	+	+	+	+	+	+	+	+	+	+	+	+	+	+	+
	KNPIAAAIGDKDG	+	+	+	+	+	+	+	+								+			+	+
	RKVLGAITGLIGDAV					+	+	+	+			+	+	+	+		+	+	+		
BBK07	SEKITKLTPEELENLAK	+	+		+	+	+	+	+	+	+		+	+	+		+	+	+		
FlaB	VQEGVQQEGAQQP	+	+					+	+	+	+	+	+	+	+	+	+	+	+		
OspC	KAILTTDAAKDKG		+						+		+									+	+
	NSVKELTSPVVVES		+					+	+				+		+				+		
Bdr*	DLENLEKQFDIKFD	+	+		+			+	+	+	+								+	+	+
BBO03^	GITKIKEEFDKKVAEIKA	+	+					+	+	+	+	+	+				+				
DbpA	GVNFDAFKDKKTGSG		+		+					+	+	+	+				+				
BBK32	EMKEESPGLFDKGNSILE		+		+		+		+		+						+		+		
BBI38	QTLSKEKAEELLQHAE	+	+		+					+	+									+	+
OspE	RKEKDGIETGLNAG		+		+				+		+		+							+	+
P37	LNVCYTDAIAALAKAKN			+	+	+				+	+										

^+^Indicates signal intensity on the serochip; NB# represents individual patient with neuroborreliosis; *Cp-32-encoded Bdr paralog family; ^Cp-32 encoded representative of paralog DUF228 family.

### Babesiosis

**Figure 2:**
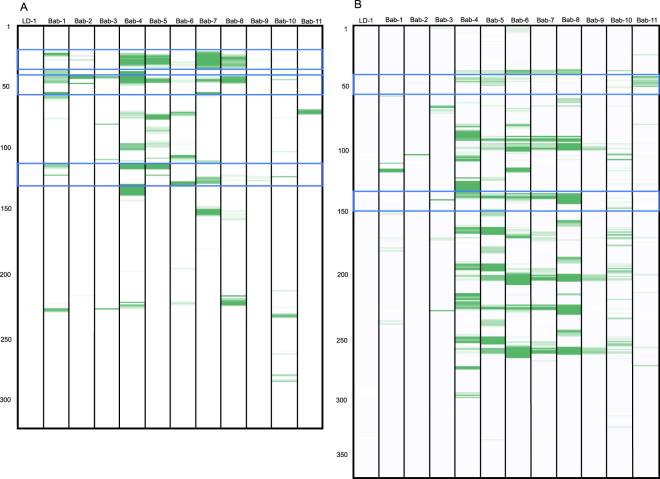
Identification of discriminatory epitopes for diagnosis of babesiosis. Shown are heatmaps for SA-1 (**A**) and BMN1–17 (**B**) antigens. The numbers on the Y-axis represent the location of 12-mer peptides positioned along the contiguous protein sequence of each antigen. Immunoreactivity with the 12-mer peptides is indicated in green with increasing signal intensity displayed from light to dark. Blue brackets indicate the range of the discriminatory epitopes from Table 3. Bab-1 to Bab-11 represent *B. microti*-antibody positive sera, LD-1 represents Lyme disease positive serum.

We tested 11 *B. microti*-antibody positive sera and identified six unique specific epitopes (three within SA-1, two within BMN 1–17 and one on BMN 1–3) (Table 3, Fig. 2). Ten of these sera also previously tested positive for *B. burgdorferi* by the two-tiered testing algorithm and all 10 were positive for *B. burgdorferi* by the TBD-Serochip, highlighting the utility of this platform for simultaneous detection of concurrent or past infections with >1 agent. In the remaining 46 Lyme disease-positive and 15 Lyme disease-negative sera, we detected *B. microti* antibodies in 4 out of 46 Lyme disease sera (9%), and in 1 out of 15 Lyme negative sera. None of these samples were previously tested for babesiosis.

### Anaplasmosis

**Figure 3:**
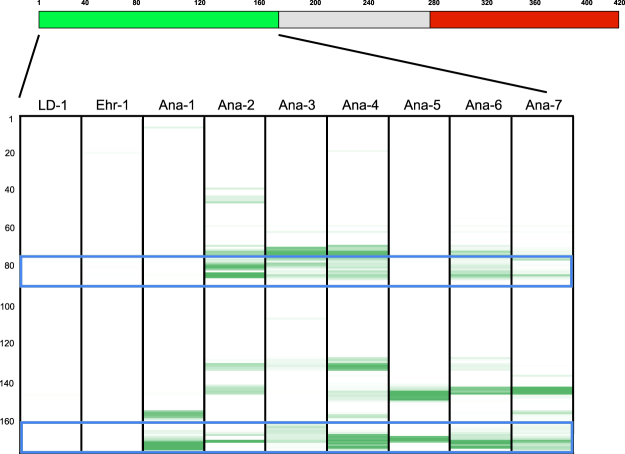
Identification of *A. phagocytophilum*-specific epitopes on MSP2. On top is a schematic of the MSP2 antigen (corresponding to accession number WP_044108210) indicating the approximate lengths of the conserved N (green) and C (red) terminal regions flanking the central variable core (gray). The heatmap displays in green the immunoreactivity of the 176 aa N terminal region, with increasing signal intensity displayed from light to dark. The numbers on the Y-axis represent the location of 12-mer peptides positioned along the contiguous sequence of the N terminal region. Blue brackets indicate the location of the discriminatory epitopes from Table 3. Ana-1 to Ana-7 indicate anaplasmosis-positive sera, LD-1 and Ehr-1 indicate Lyme disease and ehlichiosis sera, respectively.

We tested 7 sera from patients diagnosed with anaplasmosis. We identified highly reactive peptides on major surface protein 2 (MSP2, also referred to as P44). Peptides from MS3-MS5 had limited reactivity and no diagnostic utility in our assay. MSP2 consists of conserved N and C termini surrounding a highly variable central core. All seven samples were reactive with multiple epitopes within the N terminus, with epitope TSGKDIVQFAKAVEIS flanking the variable region reactive in every sample tested (Fig. 3). The reactivity was specific to *A. phagocytophilum*, and no reactivity was detected when we tested serum from a patient with an *E. chaffeensis* infection, a tick-transmitted pathogen that frequently cross-reacts in tests for anaplasmosis. In addition, five of these seven samples had antibodies to another TBD-agent, with three sera positive for *B. burgdorferi* and two for *B. microti*. Four out of 56 Lyme disease sera were also reactive to these *A. phagocytophilum* specific epitopes.

### Powassan virus

**Figure 4:**
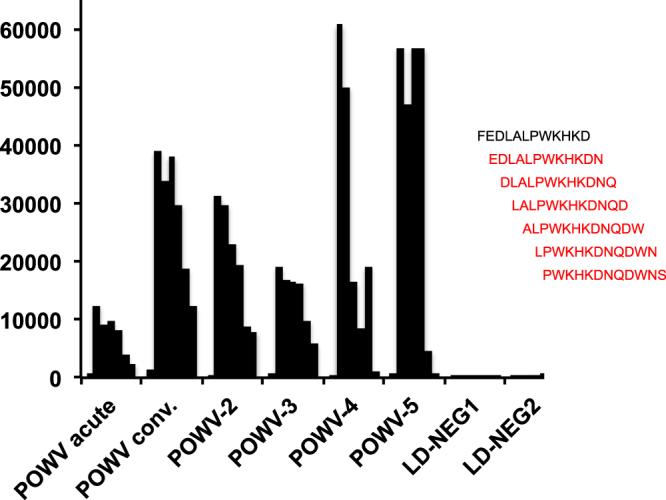
Identification of a POWV-specific peptide on the TBD-Serochip. Shown is the intensity of the fluorescent signal (indicated on the Y axis) for the seven peptides to the right. Red indicates reactive 12-mer peptides. LD-NEG corresponds to negative control samples for Powassan virus.

We tested six POWV-positive samples, consisting of four convalescent sera and paired acute and convalescent sera. All samples reacted with a specific epitope within the glycoprotein of lineage II of POWV (deer-tick virus) (Fig. 4). Three of the 56 Lyme disease sera, none with a reported history of POWV infection, had reactivity to this epitope.

### CDC validation set

We next tested a set of 32 samples available from the CDC that are used for validation of novel diagnostic platforms^52^. This set (Research I panel) contains Lyme disease positive sera (early acute with erythema migrans, disseminated, and late disease), Lyme disease-negative sera (healthy residents of Lyme disease-endemic and non-endemic regions) and sera from individuals who have no history of Lyme disease but have other diseases (infectious mononucleosis, fibromyalgia, multiple sclerosis, rheumatoid arthritis, syphilis and severe periodontitis) that may cause serologic cross-reactivity in antibody-based tests for Lyme disease. The analysis of these samples indicated that the TBD-Serochip can offer improvement in performance relative to the two-tiered algorithm for the detection of early Lyme disease (Supplementary Table 3). In addition, the results of this analysis allowed us to further streamline the list of key epitopes into the panel shown in Table 3.

## Discussion

The reported incidence of TBDs has risen continuously over the past three decades in association with geographic expansion of tick populations and the discovery of a wide range of novel tick-borne pathogens^53,54^. Nonetheless, the true incidence of TBDs is likely greatly underestimated, as patients with presumed TBDs are rarely tested for the full range of tick-borne agents, and only a fraction of positive cases are properly reported^4,55,56^. New diagnostic assays that can detect infections with the full range of TBD agents are urgently needed^15,57–59^.

The performance of the TBD-Serochip demonstrated that it is a promising tool for differential diagnosis of TBDs. The continued development of this test will require experimental comparisons to serologic assays currently considered the gold standard for TBD diagnosis. The TBD-Serochip can offer several advantages over these platforms. First, its multiplexed format screens for multiple agents as opposed to the single agent test in ELISA, WB and IFA. This is a substantial advancement, since *Ixodes scapularis* ticks alone can transmit at least five human pathogens and individual ticks are frequently infected with more than one agent. Evidence of exposure to other tick-borne pathogens in patients with Lyme disease has been well documented^60–63^. The presence of antibodies to multiple agents can be attributed to concurrent infections as well as asymptomatic, or past infections. The capacity of the TBD-Serochip to simultaneously detect both IgG and IgM can be helpful in extrapolating infection status. In our analysis of sera from patients with TBD (excluding the 10 known dual Lyme disease and babesiosis positive sera), we identified antibodies to another agent in 26% of specimens. These findings, as well as recent work by Curcio and colleagues that revealed 29% of Lyme disease patients had antibodies to *B. microti* emphasize the critical need for a multiplex test for TBDs^64^.

Early detection of infection enables rapid and appropriate treatment, thereby reducing morbidity. Here too, the TBD-Serochip can improve on the current tests used in diagnosis. We identified a wide range of specific peptides, including the first discriminatory epitopes for anaplasmosis and Powassan disease, as well as new diagnostic targets for Lyme disease and babesiosis. The application of these peptides in concert can enhance the likelihood of antibody detection in early disease specimens. The TBD-Serochip also allows simultaneous detection and confirmation of infection. Whereas the current diagnostic algorithm for diagnosis of Lyme disease requires ELISA followed by western blots, the TBD-Serochip can provide evidence of immunoreactivity to multiple epitopes in a single reaction. Discriminatory epitopes identified through TBD-Serochip analyses can also readily be adapted to other serologic platforms, such as ELISA, Luminex or lateral flow immunoassays.

As new TBD agents are discovered, the TBD-Serochip is inherently more flexible than other platforms because new targets can be added to arrays through *in situ* peptide synthesis. Target protein selection, peptide design, and manufacture of a TBD-Serochip can be executed in less than 4 weeks, facilitating rapid development of new and specific diagnostic tests for novel TBDs agents.

In addition to its utility as a diagnostic platform, the TBD-Serochip provides a powerful research tool for studies of TBDs. The TBD-Serochip can be employed to discriminate individual antibody responses in patients with TBD and thus examine the interplay of TBD agents on disease manifestation and progression. It can also be used to assess the impact of genetic diversity of tick-borne pathogens on the host immune response. The array can resolve antibodies to different genotypes of TBD agents, such as OspC types of *B. burgdorferi*, or analyze the temporal response to the multitude of *B. burgdorferi* VlsE or *A. phagocytophilum* MSP2 antigenic variants generated during the course of disease. It can also be utilized to identify potential changes in reactive epitopes in individuals with persistent symptoms following antibiotic treatment.

A limitation of this platform is that we are only displaying linear peptides. While this does not appear to confound the diagnostic utility, we may miss conformation determinants or non-protein epitopes important in pathogenesis. We have not included known targets of autoimmunity; however, these could easily be addressed with linear peptides.

## Methods

### Human samples

De-identified human sera and cerebral spinal fluid (CSF) samples were acquired from the New York State Department of Health, State University of New York at Stony Brook, the Lyme and Tick-Borne Disease Research Center at Columbia University, Long Island University, National Institutes of Health and the Centers for Disease Control and Prevention. Sample types ranged from early to late Lyme disease (confirmed by the two-tiered testing algorithm), non-Lyme TBDs (babesiosis, anaplasmosis, ehrlichiosis and Powassan virus disease), and non-Lyme controls (Lyme ELISA-negative). All anaplasmosis and babesiosis samples were retested at the Center for Infection and Immunity by IFA (Fuller Labs, Ca) prior to the TBD-Serochip analysis. For selected Lyme disease-positive sera, we performed the C6 ELISA (Gold Standard Diagnostics) according to manufacturers instructions. Paired sera and cerebrospinal fluid samples from patients with acute Lyme neuroborreliosis were obtained under a clinical protocol (ClinicalTrials.gov Identifier: NCT00028080) approved by the institutional review board of the National Institute of Allergy and Infectious Diseases, and all patients signed informed consent. All methods used for sample collection were performed in accordance with the proper guidelines and regulations.

### Data acquisition

Samples were diluted in binding buffer (0.1 M Tris-Cl, 1% alkali soluble casein, 0.05% Tween-20) and hybridized to the arrays at 4 °C for 14 hours. Arrays were washed 3 times for 10 minutes each on Little Dipper (SciGene) with 1X TBST (0.05% Tween-20) at room temperature. Secondary antibodies were diluted in binding buffer at concentration of 1ug/ml. Secondary antibody incubation was done in Plastic Coplin Jar (Fisher Scientific,) for 3 hours at room temperature with gentle agitation. To enable simultaneous detection of both IgM and IgG antibodies, we used a secondary antibody incubation method where Alexa Fluor 647-AffiniPure Goat Anti- Human IgG, Fc-γ fragment specific (Jackson ImmunoResearch Labs, Inc) was first bound to the array, then washed 3 times (10 minutes each) on Little Dipper with 1X TBST at room temperature and spun dry. This was followed by binding Cy3-conjugated AffiniPure Fab fragment Goat Anti-Human IgM, Fc5_μ_ Fragment (Jackson ImmunoResearch Labs, Inc), repeating the same washing and drying methods. Each secondary antibody incubation was followed by 3 washes (10 minutes each) on Little Dipper with 1X TBST at room temperature and spin drying. The slides were scanned on NimbleGen MS 200 Microarray Scanner (Roche) at 2 μm resolution, with an excitation wavelength of 532 nm for Cy3/IgM and 635 nm for Alexa Fluor/IgG, respectively.

### Data analysis

The relative fluorescent unit (RFU) signals for all the probes were extracted from the images using Roche Sequencing Solutions image extraction software. The RFU signals were converted into intensity plots after quantile normalization, background and spatial correction, and deconvolution for redundant peptides. To determine background noise, we obtained signal intensity data for each antigen from 26 control serum and 20 cerebrospinal fluids (CSF) samples found to be negative for all agents tested. A reactive epitope was identified by a a continuous set of overlapping 12-mer peptides with signal intensities above threshold in samples with known TBDs. The signal threshold was defined for each reactive epitope by calculating the mean plus 3 standard deviations of the signal intensity for the same epitope in the 26 negative control samples. An epitope was considered reactive when the signal intensity for at least two contiguous 12-mer peptides was above threshold. An individual sample was called positive when reactive with at least one specific epitope.

### Data availability

The datasets generated during and/or analyzed during the current study are available from the corresponding author on reasonable request.

### Disclaimer

This research was supported in part by the Intramural Research Program of the NIH, NIAID. The content of this publication does not necessarily reflect the views or policies of the Department of Health and Human Services, nor does mention of trade names, commercial products, or organizations imply endorsement by the US government.

## Electronic supplementary material

Dataset 1
