# Supplementary material for: A multiplex serologic platform for diagnosis of tick-borne diseases
Source: Sci Rep. 2018 Feb 16;8:3158. doi: 10.1038/s41598-018-21349-2 (PMC5816631; doi:10.1038/s41598-018-21349-2)
Supplement: Supplementary file 1 — Dataset 1 [file 41598_2018_21349_MOESM1_ESM.pdf]

Supplementary Information

A multiplex serologic platform for diagnosis of tick-borne diseases

Rafal Tokarz, Nischay Mishra, Teresa Tagliafierro, Stephen Sameroff, Adrian Caciula, Lokendrasingh Chauhan, Jigar Patel, Eric Sullivan, Azad Gucwa, Brian Fallon, Marc Golightly, Claudia Molins, Martin Schriefer , Adriana Marques, Thomas Briese, W. Ian Lipkin

Supplementary Table 1. List of early Lyme disease samples analyzed on the TBD-Serochip

Sample	WB IgM	WB IgM bands	WB IgG	WB IgG bands	C6 index CII	VlsE	FlaB	OspCNt	OspC type K	OspC type M	P100	BBK07	Bdr	OppA	BBO03
RTS-900	POS	2 BANDS	NEG	0 BANDS	.34									+	
RTS-901	POS	2 BANDS	NEG	0 BANDS	1.38							+	+	+	
RTS-902	POS	3 BANDS	NEG	0 BANDS	7.78	+						+			
RTS-903	POS	3 BANDS	NEG	1 BAND	7.39	+	+								
RTS-904	POS	2 BANDS	NEG	1 BAND	1.3		+		+						
RTS-905	POS	2 BANDS	NEG	1 BAND	1.15									+	
RTS-906	POS	2 BANDS	NEG	1 BAND	0.25										+
RTS-907	POS	2 BANDS	NEG	1 BAND	8.2	+									
RTS-908	POS	2 BANDS	NEG	1 BAND	1.4	+									
RTS-909	POS	2 BANDS	NEG	2 BANDS	1.51	+	+	+							
RTS-910	POS	2 BANDS	NEG	2 BANDS	3.61	+						+			
RTS-911	POS	2 BANDS	NEG	3 BANDS	N.D.	+						+	+		
RTS-912	POS	2 BANDS	NEG	4 BANDS	2.38	+						+			
RTS-201	POS	*na*	NEG	*na*	ND	+	+								
RTS-600	POS	*na*	NEG	*na*	ND	+	+		+			+		+	
RTS-602	POS	*na*	NEG	*na*	0.61								+		
RTS-604	NEG	*na*	NEG	*na*	.39							+			
RTS-606	POS	*na*	NEG	*na*	ND						+				+
RTS-607	POS	*na*	NEG	*na*	ND	+									
RTS-609	POS	*na*	NEG	*na*	ND	+									
RTS-614	POS	*na*	NEG	*na*	.99			+			+				
RTS-615	POS	*na*	NEG	*na*	ND				+						
RTS-619	POS	*na*	NEG	*na*	4.8							+			+
RTS-620	POS	*na*	NEG	*na*	2.97	+		+	+						
RTS-625	POS	*na*	NEG	*na*	ND	+									
RTS-628	POS	*na*	NEG	*na*	ND	+									
RTS-633	POS	*na*	NEG	*na*	ND	+		+							

na - data not available; NEG - negative; ND - not done; + indicates TBD-Serochip signal intensity above threshold

Supplementary Table 2. List of IgG positive Lyme disease samples analyzed on the TBD-Serochip

Sample	WB IgM	WB IgM bands	WB IgG	WB IgG bands	C6 index CII	VlsE	FlaB	OspC Nt	OspC type K	OspC Type M	P100	BBK07	Bdr	OppA	BBO03
RTS-913	IND	1 BAND	POS	6 BANDS	4.68	+						+			
RTS-914	NEG		POS	6 BANDS	4.8	+									
RTS-915	NEG		POS	7 BANDS	ND	+	+					+			
RTS-916	IND	1 BAND	POS	7 BANDS	ND	+									
RTS-0202	NEG	*na*	POS	*na*	ND	+	+		+						
RTS-0204	POS	*na*	POS	*na*	ND	+	+								
RTS-0205	POS	*na*	POS	*na*	ND	+				+					
RTS-0206	POS	*na*	POS	*na*	ND	+	+					+			+
RTS-0207	POS	*na*	POS	*na*	ND	+	+					+			
RTS-0208	POS	*na*	POS	*na*	ND	+	+		+						
RTS-0209	POS	*na*	POS	*na*	ND	+	+					+			
RTS-0210	POS	*na*	POS	*na*	ND	+	+					+			+
RTS-0211	POS	*na*	POS	*na*	ND	+	+					+			
RTS-0212	POS	*na*	POS	*na*	ND	+	+					+			
RTS-0213	POS	*na*	POS	*na*	ND	+	+					+			
RTS-0219	NEG	*na*	POS	*na*	ND	+	+						+		+
RTS-0220	POS	*na*	POS	*na*	ND	+	+					+			
RTS-655	POS	*na*	POS	*na*	10	+	+								+
RTS-659	NEG	*na*	POS	*na*	ND	+	+		+			+			

na - data not available; NEG - negative; ND - not done; + sign indicates TBD-Serochip signal intensity above threshold

Supplementary Table 3. Reactivity of discriminatory epitopes for *B. burgdorferi* with Lyme disease positive sera from the CDC Research panel I

Sample Category	Sample Group	Acute/ Convalescent	EIA	IgM WB	IgM WB bands	IgG	IgG WB Bands	2-Tier Interpretation	VlsE	FlaB	OspC Nt	OspC type K	OspC type M	P100	BBK07	Bdr	OppA	BBO03
Lyme disease	Early Lyme-EM	Acute	Neg	Neg	-	Neg	1	Neg	+									
Lyme disease	Early Lyme-EM	Acute	Neg	Neg	-	Neg	1	Neg										+
Lyme disease	Early Lyme-EM	Acute	Neg	Neg	-	Neg	2	Neg	+									
Lyme disease	Early Lyme-EM	Acute	Neg	Pos	2	Neg	2	Neg			+			+			+	+
Lyme disease	Early Lyme-EM	Convalescent	Pos	Pos	2	Neg	2	Pos	+	+					+		+	
Lyme disease	Early Lyme-EM	Convalescent	Pos	Pos	2	Neg	4	Pos	+				+					+
Lyme disease	Early Lyme-EM	Convalescent	Pos	Pos	2	Pos	5	Pos	+			+						
Lyme disease	Early Lyme-EM	Convalescent	Pos	Pos	3	Neg	3	Pos	+		+			+				
Lyme disease	Neurologic Lyme	-	Pos	Pos	3	Pos	5	Pos	+	+	+			+				
Lyme disease	Neurologic Lyme	-	Pos	Pos	2	Pos	10	Pos	+	+					+			+
Lyme disease	Lyme arthritis	-	Pos	Neg	1	Pos	7	Pos	+	+				+	+			+
Lyme disease	Lyme arthritis	-	Pos	Neg	1	Pos	10	Pos	+	+					+			

\+ indicates TBD-Serochip signal intensity above threshold

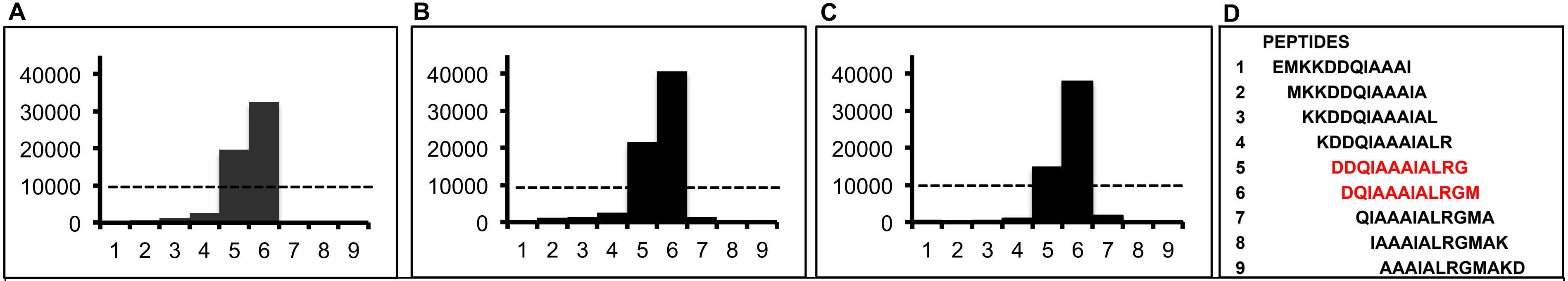

Supplementary Figure 1. Reproducibility of the TBD-Serochip. Shown are the data for the C6 region of the VlsE protein of *B. burgdorferi.* Panels A and B represent results from an early acute Lyme disease sera tested twice on the same serochip. Panel C shows the results from the same sample run on a different serochip. Y axis represents the intensity of the fluorescence signal, X axis represents 9 overlapping peptides within the C6 (panel D). Significant immunoreactive peptides are indicated in red. Dashed lines represent the threshold for the MKKDDQIAAAIALRGMA epitope
